# A systematic review and meta-analysis of group peer support interventions for people experiencing mental health conditions

**DOI:** 10.1186/s12888-021-03321-z

**Published:** 2021-06-23

**Authors:** Natasha Lyons, Chris Cooper, Brynmor Lloyd-Evans

**Affiliations:** 1 Division of Psychiatry, University College London, Maple House, 149 Tottenham Court Road, London, W1T 7NF UK; 2 Department of Clinical, Educational and Health Psychology, University College London, London, WC1E 7HB UK

**Keywords:** Peer support, Group interventions, Mental health services, Systematic review, Meta-analysis, Recovery

## Abstract

**Background:**

Peer support is being integrated within mental health services to further the development of a recovery approach. However, the most effective models and formats of intervention delivery are unknown. We conducted this systematic review and meta-analysis to determine the effectiveness of peer support for improving outcomes for people with lived experience of mental health conditions, when delivered as group interventions.

**Methods:**

Studies reporting randomised controlled trials of group peer support interventions for people experiencing mental health conditions were identified by searching MEDLINE, PsycINFO, Embase and Cochrane CENTRAL, from inception until July 12th 2019 and undertaking supplementary searches. Included studies were assessed for risk of bias and meta-analyses were conducted if three or more trials provided usable data.

**Results:**

Eight trials met eligibility criteria, providing data from 2131 participants. Six trials had either high or unclear risk of bias. Interventions were categorised as mutual support groups, or peer support groups, sub-categorised as anti-stigma or self-management interventions.

Meta-analyses were only possible for peer support groups and five outcomes. We found evidence that group peer support may make small improvements to overall recovery but not hope or empowerment individually, or to clinical symptoms. Evidence for effectiveness for outcomes which could not be meta-analysed was mixed.

**Conclusions:**

Findings from the few eligible trials suggest group peer support interventions may be specifically effective for supporting personal recovery and have a limited impact on other outcomes, though there were some risks of bias to study findings. Interventions were heterogeneous and most social outcomes were absent in the literature, highlighting further limitations to the current evidence-base. There is insufficient evidence available from trials of group peer support torecommend the routine implementation of these interventions across mainstream mental health services at present. More high-quality trials of peer-developed, group peer support interventions are needed in order tomake firm conclusions about intervention effectiveness.

**Supplementary Information:**

The online version contains supplementary material available at 10.1186/s12888-021-03321-z.

## Background

Transition to a recovery approach is a key focus of national [1] and international [2] mental health service development. Peer support has been characterized as a truly recovery-orientated intervention [3] and is now recommended in policy guidance internationally [4–6]. This reflects a growing recognition of the value of lived experience expertise for facilitating recovery within mainstream services [7]. Peer support enables individuals with personal experience of mental health conditions to utilise this experiential expertise to assist people accessing mental health services with the process of recovery [8]. Support may be unidirectional, such as from a paid peer support worker to a recipient, or reciprocal, as in mutual support groups [9]. Interventions involving unidirectional support have been further categorised as: peer support services, delivered alongside traditional providers; or peer-delivered services, delivered by peers as alternative providers to non-peer professionals [8]. Peer-delivered services tend to be complex interventions, and peer support services and mutual support may be delivered as one-to-one or group interventions [10].

The distinct therapeutic processes that distinguish group and individual peer support approaches are not yet clearly defined, which reflects the lack of consensus on the broader mechanisms of peer support [11, 12]. Reviews of proposed mechanisms [11, 12] suggest that recovery may be enhanced through personal identification and modelling of positive social behaviours [11, 13], “upward” social comparisions [14] with recovery role models and through the exchange of experiential knowledge [11, 15]. Experiential learning may lead to the development of an alternative knowledge base for mental health management based on individual realities of recovery [16, 17]. Social support has been proposed to operate within peer relationships [18] through the exchange of emotional and informational resources between individuals [19]. A group setting may therefore maximise the potential for exchange of recovery resources and opportunities for experiential learning.

In spite of the potential for group peer support to improve recovery, only one review to date, has focused specifically on the effectiveness of group peer support approaches and this solely included mutual support groups [20]. This review was published over 10 years ago and synthesised studies with both randomized and non-randomized designs [20]. Studies included in this earlier review reported mixed evidence for improving clinical outcomes, such as psychiatric symptoms [20]. This contributes to the mixed evidence-base for peer support in general, though considerable risks of bias to study findings often reduce confidence in the available evidence [21]. Previous reviews of peer support have often focused on interventions for participants with particular diagnoses [22, 23], which may mask further transdiagnostic benefits based on shared experiences of mental health conditions and of using mental health services [24].

Across reviews, current evidence suggests that peer support may have particular effectiveness for improving outcomes related to personal recovery [9] as opposed to clinical outcomes [21, 25]. Where it has been possible to isolate the effects of group peer support within reviews, specificity for enhancing personal recovery has similarly been suggested, including improvements to hope [25] and empowerment [10] outcomes but not clinical symptoms [25]. Two descriptive reviews have also indicated positive effects on both clinical and recovery outcomes for peers-delivering educational curricula in group format [26] and mutual support groups [8].

With the continued international expansion of peer support within mental health services [27] and increasing research focus on peer support interventions [22], there is a pressing need to update to the evidence for effectiveness from previous reviews. The heterogeneity in peer support interventions has led to a call for a greater focus on specific effectiveness with respect to categorisations and contexts [26]. Determining the optimum format of intervention delivery is needed to inform the implementation of peer support within service developments and the specific effectiveness of group peer support has not been fully addressed. Recommendations guiding implementation are currently hampered by conflicting findings within the literature with respect to the relative effectiveness of group and one-to-one peer support for improving personal recovery outcomes, with one review reporting more evidence to support individual [25] and another, for group [10] approaches. Although the more recent review [10] synthesised evidence for the effectiveness of group peer support for empowerment and self-efficacy, consideration of a broader range of outcomes may contribute to a holistic appraisal of intervention effectiveness for recovery outcomes. Therefore, this review aims to narratively and quantitively synthesis evidence from randomised controlled trials (RCTs) for the effectiveness of group peer support for improving outcomes for people with mental health conditions, compared to any comparator condition; including outcomes relevant to personal and clinical recovery [28], acute service-use and social indicators of recovery, such as social support [29] and employment [30]. Our review complements a review of one-to-one peer support interventions carried out contemporaneously byWhite and colleagues at St George’s University [31]. Findings for group peer support will be discussed in the context of current evidence regarding one-to-one peer support.

## Methods

The research methods of this review were conducted in accordance with the Cochrane Collaboration’s guidelines for systematic reviews of interventions [32] and reported following the Preferred Reporting Items for Systematic Reviews and Met-Analysis (PRISMA) statement [33] (the PRISMA checklist for each item is included in Additional file 2). The protocol for the review was prospectively registered on PROSPERO, International Prospective Register of Systematic Reviews, registration number: CRD42019145217.

### Study identification

Studies were identified using both bibliographic database searching and non-bibliographic search methods [34].

#### Bibliographic databases

We searched the following bibliographic databases from inception: PsycINFO, MEDLINE, Embase (all via the OVID interface) and the Cochrane Central Register of Controlled Trials (CENTRAL) via the Wiley interface. Search terms were developed and piloted in PsycINFO, then adapted for use on the other databases. In order to pilot our search terms, we first identified “model” papers, which included clear examples of group peer support interventions. We identified these from an initial google and bibliographic database search for studies and reviews of peer support interventions for people who experience mental health conditions. Search terms were then revised and refined, to maximise the relevancy of the search results and to ensure all model papers were returned. The Peer Review of Electronic Search Strategies (PRESS) checklist was used to peer-review the search strategy prior to searching [35].

The search strategy adopted the structure: (search terms for peer support, such as “peer-led” or peer* adj3 support*) AND (all fields group search) AND (RCT search filter). The Cochrane Highly Sensitive Search Strategy was used in MEDLINE [36] and the Royle and Waugh filter [37], supplemented with the P3 filter to maximise sensitivity [38], for PsycINFO and Embase. No language limits were applied to the searches. The full search strategy is included in the supplementary material for this review (Additional file 1). The MEDLINE search is reported with a search narrative which explains the conceptual and contextual detail of the design of the search strategy [39].

#### Non-database search methods

The following non-database search methods were used:

- Two trial registers were searched: ClinicalTrial.gov and the World Health Organization International Clinical Trials Registry platform
- Citation searching on all studies meeting inclusion at full-text was undertaken. Forwards citation searching was undertaken in Web of Science and backwards citation chasing searching was undertaken manually by appraisal of the reference list of included studies
- The list of included studies was manually reviewed for any systematic review identified by the searches
- For any protocols returned by the searches, or any on-going trials identified by the trial registers, the corresponding authors of the study were contacted to establish if their studies had completed and if unpublished data were available.

The first 10% of all records were independently screened by two reviewers. Inter-rater agreement was 100% at this stage so the remaining abstracts were screened by one reviewer. The full text articles of potentially eligible articles were retrieved and assessed for eligibility for inclusion by one reviewer (NL). The second reviewer (CC), blind to the first reviewer’s screen, then screened all included studies and 10% of the excluded studies, to check for concordance. A third researcher (BLE) was involved to resolve any disagreements regarding inclusion. If this failed to resolve discrepancies, study authors were contacted for further clarification.

### Eligibility criteria

#### Study design

We included only completed RCTs with individually randomised designs. Published and unpublished, completed trials were eligible for inclusion. Cluster RCTs, incomplete RCTs and all non-randomised designs were excluded, including partially randomised and quasi-experimental designs.

#### Participants

Eligible participant populations were adults aged 18 and over with mental health conditions. Participants were identified as having confirmed mental health conditions if they met one or more of the following three criteria:

1. Use of mental health services, defined as a statutory or voluntary sector service that provides support exclusively for people with mental health conditions.
2. A clinical diagnosis of any condition within the International Classification of Diseases axis 1 psychiatric disorders, which includes common mental health conditions, such as depression and anxiety disorders, those defined as severe, such as bipolar and schizophrenia spectrum disorders, and other mental health conditions including personality disorders, eating disorders and dissociative disorders.
3. Assessed as experiencing psychiatric symptoms reaching a clinical threshold using any validated symptom rating tool.

Studies were excluded if they included only participants with organic neurological pathologies such as dementia, ordisorders typically diagnosed in childhood, such as conduct disorder, or developmental disorders such as autism, or alcohol or substance misuse related disorders.

#### Interventions

We included studies of intentional, group peer support interventions, delivered solely by and to people with mental health conditions. Interventions were only included if the primary focus was to promote recovery with mental health conditions. Recovery was broadly defined as “ … a deeply personal, unique process of changing one’s attitudes, values, feelings, goals, skills, and/or roles. It is a way of living a satisfying, hopeful, and contributing life even with limitations caused by [mental health conditions].” [40] (p257)

Both mutual support groups and peer-facilitated, peer support services delivered in group format were included. Only interventions intended for more than two participants were included.

One-to-one peer support interventions and complex interventions involving group and individual peer support were excluded. We also excluded interventions co-facilitated, facilitated or guided by health professionals. Group peer support interventions were excluded if the focus was any topic other than recovery with mental health conditions, including bereavement and physical health conditions, even if participants in these groups had mental health conditions. Interventions with a primary focus on recovery from addiction were also excluded. This is because these interventions aim to provide support to reduce or achieve abstinence from addictive behaviours as part of recovery [41], which may necessitate unique characteristics and approaches. There are a large number of active peer-led and mutual support organizations that promote recovery programs, with an independent evidence-base [20] that is outside the scope of this review.

We did not exclude any studies based on control condition and included studies that compared group peer support with treatment as usual (TAU), however defined, or a waiting list control or with any active control intervention.

#### Outcomes

We included studies that reported any of the broad groups of outcomes below, however measured:

1. Personal Recovery

Studies reporting any measure of recovery were included. We also included studies reporting any outcome defined as a component of recovery by the CHIME framework [42]. This acronym refers to connectedness, such as relationships, hope, identity, meaning and empowerment. Studies reporting self-esteem, personal confidence, self-efficacy and quality of life were also included.

2) Clinical Recovery

We included studies reporting clinical outcomes, such as any measure of psychiatric symptoms, including symptom scale ratings or clinical recovery rates, and any clinical measure of social functioning.

3) Acute mental health service use

Studies that reported any measure of acute mental health service use, such as number of hospital admissions, crisis care admission or inpatient bed days, were included.

4) Social outcomes

We included studies reporting the following outcomes: employment (voluntary or paid), independent living (defined as supported or independent accommodation type) and social support (measures of social network or other social support within the community).

### Risk of bias assessment

The first reviewer (NL) conducted a risk of bias assessment for each included study using the Cochrane Collaboration’s Tool for assessing bias in randomised trials [43]. This included assessment of random sequence generation, allocation concealment, blinding of participants, researchers and of outcome assessors, completeness of outcome data and selective outcome reporting. Each domain of bias was rated as low, high or unclear risk of bias (ROB), according to the guidance specified by the tool and the Cochrane Handbook [44], indicating whether each form of bias was unlikely or highly likely to have influenced study outcomes or may have influenced study outcomes but insufficient information was reported to make a judgement, respectively. A random sample of 10% of studies were assessed by the second reviewer (CC) using the same procedure. Any disagreements were resolved through discussion with a third researcher (BLE).

Studies that were rated as low ROB in every domain of bias were categorised as low overall ROB [44]. Selection bias (random sequence generation and allocation concealment) and risks fromincomplete reporting of study data (attrition bias and reporting bias) were considered key risks of bias [43], so studies with high or unclear risks in these domains received these ratings overall. These ratings indicate the likelihood that bias influenced the overall findings of the study.

### Data extraction

The Cochrane Collaboration data extraction form for RCTs was adapted and piloted with three of the included records prior to use. Data extracted from eligible studies included: study aims, study setting, study duration, participant eligibility criteria, total number of participants randomised, participant characteristics including age, gender, ethnicity and mental health diagnoses, baseline imbalances, details of attrition, intervention and control group characteristics, missing outcome data and the results of the outcomes measured at all time points recorded. Raw means and standard deviations and number of participants providing data for each outcome were extracted for the quantitative synthesis.

### Statistical analysis

Meta-analyses using random effects models were conducted for outcomes where possible, using Review Manager (RevMan 5.3) software [45]. For the main analysis, meta-analyses were conducted separately for each outcome within the broad outcome groups. For example, within the recovery outcome group, studies reporting empowerment were analysed together. Studies that used TAU or active controls were analysed together for the main analysis, by combining the means and standard deviations for TAU and active comparators using the formulae recommended by the Cochrane Handbook [46].

All outcomes were categorised by timepoint as post-intervention (recorded at the end of treatment), short-term follow-up (up to 1 year after the end of treatment) and long-term follow-up (more than 1 year after the end of treatment). If outcome data at multiple time points were reported by studies, the timepoint nearest to but not exceeding one-year follow-up was used for short-term follow-up, and the longest duration of follow-up was used for long-term follow-up. Outcomes at each timepoint were analysed separately. All studies that reported an outcome and provide usable data were included in the main analyses for each outcome, regardless of study population, intervention type or ROB rating but we set three studies as a minimum number to perform any meta-analysis. The inverse variance method wasused to calculate standardised mean differences (SMD) for continuous outcomes using different outcome measures and the magnitude of this effect size (Cohen’s*d*) was interpreted as small (0.2), medium (0.5) or large (0.8) [47, 48]. For studies using the same outcome measure, mean differences were calculated. Strength of the evidence for an effect was determinedby Z statistic *p*-values and categorised as no evidence (*p* ≥ 0.1), weak evidence (*p* = 0.09–0.01), strong evidence (*p* < 0.01) and very strong evidence (*p* < 0.001) [49].

Heterogeneity was assessed using the non-central Chi^2^ method and theI^2^ statistic. We defined I^2 ^of greater than 50% as substantial heterogeneity [46]. Tests of funnel plot of asymmetry were planned for meta-analyses with ten or more studies only, since fewer than ten studies lack sufficient power to produce reliable estimations of publication bias [50].

For outcomes for which fewer than three studies provided usable data, study results were summarised and described narratively.

### Sensitivity and subgroup analyses

Two sensitivity analyses were performed to analyse studies with low overall ROB separately from those with unclear and high ROB and to analyse studies using TAU comparators separately from those using active controls.

Two planned subgroup analyses were undertaken. First, interventions for people with mental health experiences defined as severe mental health conditions were analysed separately from those with other mental health conditions. The definition of severe mental health conditions used in this review included consideration of functional impairment [51] and included participants with bipolar disordersor psychosis spectrum disorders or participants with any diagnosis using secondary mental health services.

The second planned subgroup analysis was to analyse structured and unstructured peer support interventions separately. Structured interventions were defined as those using manuals or pre-defined programme plans, whereas unstructured interventions were defined as those where the content of group sessions was flexible and could be determined by the group.

## Results

The database search was conducted on the 13th of July 2019 and returned 7198 records. Supplementary searche sidentified a further 225 studies for screening. Following duplicate removal, the titles and abstracts of 4277 records were screened for eligibilityand 4179 records documenting clear exclusion criteria were excluded at this stage. Reasons for exclusion included clear evidence of ineligibility due to study type, intervention typeor study population in the title or abstract of the record. The full texts of 98 articles were retrieved and eight studies, reported by 11 articles, were included in the review. Of these, six studies provided usable data for meta-analyses. A total of 87 studies were excluded at full text screen (the full PRISMA Flow Diagram is presented in Additional file 3).

### Characteristics of included studies

Study characteristics are displayed in Table 1. All included studies were individually randomized controlled trials with parallel group designs. Seven trials took place in America and one was conducted in Switzerland. Six trials reported follow-up data [52, 53, 56, 60–62] ranging from 3 weeks to 6 months after the end of treatment.

**Table 1:** Study Characteristics

Study ID	Intervention Category	Country	N	Diagnoses	Sex % F	Ethnicity, % BAME	Age	Employed %
Ben-Zeev 2018 [52]	*Peer Support group*: Self-management	USA	163	49% SS 28% BPD 33% MDD	40	72	49	N/R
Cook 2012a [53–55]	*Peer Support group*: Self-management	USA	555	20% SS, 38% BPD, 25% DD, 15% other	66	37	46	15
Cook 2012b [56, 57]	*Peer Support group:* Self-management	USA	428	21% SS, 40% BPD, 18% DD 9% other	56	46	43	9
Eisen 2012 [58]	*Peer support group*: Self-management	USA	298	Psychotic disorders, DD, alcohol/ substance misuse disorders (% N/R)	8	33	72% were 36–60 years	N/R
Kaplan 2011 [59]	*Mutual support*: online group	USA	300	22% SS, 78% affective disorders	66	8	47	63
Corrigan 2015 [60]	*Peer Support group:* Anti-Stigma	USA	205	N/R	64	64	46	24
Rüsch 2014 [61]	*Peer Support group*: Anti-stigma	SC	100	27% SS 20% BPD 60% DD	59	2	42	19
Russinova 2014 [62]	*Peer Support group*: Anti-stigma	USA	82	34% SS 33% BPD 26% DD 7% other	68	31	68% were > 40 years	16

Ages and Inpatient admissions are reported as Means and % respectively unless otherwise stated

*USA* United States of America, *SC* Switzerland Confederation, *N* Total number of participants randomised, *SS* schizophrenia spectrum disorders, *BPD* Bipolar Disorder, *MDD* Major Depressive Disorder, *N/R* not recorded, *DD* Depressive disorder, *Other* category reported by papers, *BAME* Black Asian and Minority Ethnicity, *(F)* Female

#### Participant characteristics

A total of 2131 participants were included in the review with a median study sample size of 252 and range of 82 to 555 participants. Across trials, the median of mean participant ages was 46 years, the median proportion of female participants was 66% and the proportion of participants identifying as Black, Asian and minority ethnicities ranged from 2 to 72%. The proportion of employed participants ranged from 9 to 63%. The participant eligibility criteria of all trials included a range of mental health diagnoses. One study did not report participant diagnoses but all participants were using mental health services [60]. All seven remaining trials comprised participants experiencing psychoses and affective disorders, however, in accordance with protocol specifications for categorising participant populations with mixed mental health conditions, only five of these trials met our criteria for comprising participants experiencing severe mental health conditions [52, 53, 56, 58, 62], since all participants in these studies were using secondary mental health services.

#### Characteristics of interventions

Details of the characteristics of study interventions are summarised in the supplementary material (Additional file 1). Intervention durations ranged between 3 weeks and 12 months. Only one study [59] used an unstructured intervention and was classified as mutual support. This study adopted two unmoderated, online peer support group interventions, which were combined for the analyses and compared to TAU. One intervention was a “listserv”, enabling participants to send emails to the whole intervention group and the other was an online bulletin board, where participants could post and readgroup messages.

Seven trials used structured interventions, classified as peer support groups, delivered by one to three peer facilitators. Structured interventions were further categorised as: self-management interventions, to develop coping strategies for mental health conditions [63]; or anti-stigma interventions, to improve responses to experienced stigma and reduce self-stigmatising behaviour [64]. All structured interventions included an educational component, delivered as classes with structured topics.

Peer Support groups:

1. Self-management interventions

Four trialswere peer-led, self-management interventions [52, 53, 56, 58]. Two of the included trials were of Wellness Recovery Action Planning (WRAP) [52, 53]. One study used WRAP as the control group to assess the comparative effectiveness of FOCUS, a self-management mobile phone application [52]. Two studies compared interventions to a Waiting List Control (WLC) [53, 56] and one study used both TAU and a clinician-led group of the intervention as control groups [58].

The number of classes ranged from eight to 12 across interventions. All interventions adopted different approaches to developing and implementing recovery-focused coping strategies. These included increasing knowledge through an educational course for Building Recovery of Individual Dreams and Goals (BRIDGES) [56], use of recovery workbooks for vet-to-vet, an intervention for veterans experiencing mental health conditions [58] and development of a personalised daily and crisis management plan for WRAP [52, 53].

2. Anti-stigma interventions

Three trials were manualised anti-stigma interventions [60–62]. Two trials were studies of the three session Coming Out Proud (COP), compared to a WLC [60] or TAU [61]. The remaining trial was a study of ten sessions of photovoice compared to a WLC [62]. Group discussions for COP included support and strategies for disclosure of mental health conditions, and for Photovoice, education about mental health stereotypes and use of a camera to develop narratives about mental health and stigma.

### Risk of bias assessment

**Fig. 1:**
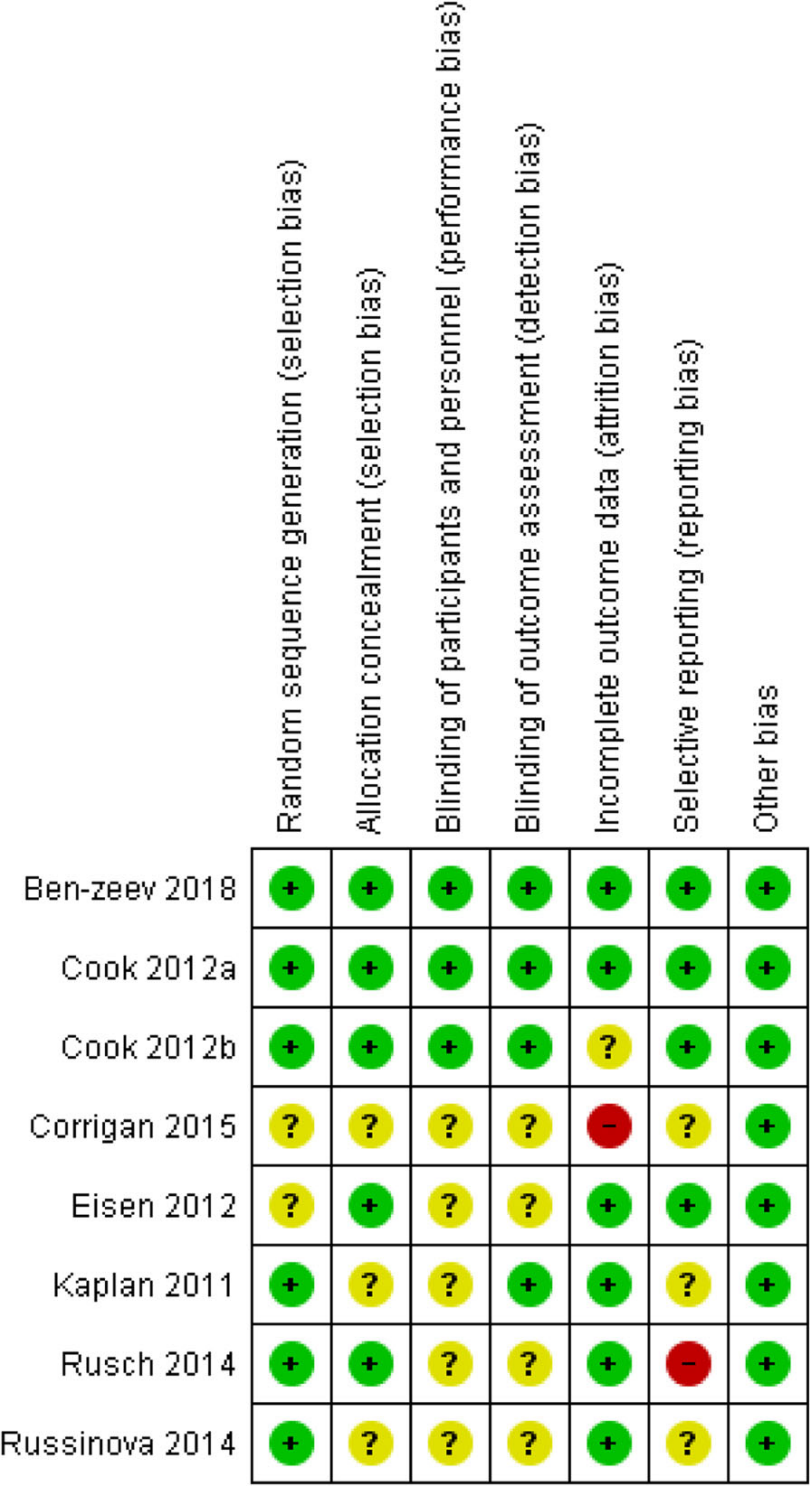
Risk of bias assessment

The ROB assessment for individual studies is displayed in Fig. 1. One study [56] had unclear risk of attrition bias since the rate of overall attrition from the study exceeded 20% [65] the participant characteristics of those who dropped out and those who remained in the study were not described and no reasons for attrition were documented. A further study [60] had high risk of attrition bias as overall attrition exceeded 20%, there was an imbalance in the numbers remaining in intervention and control groups and participant characteristics of those who dropped out and reasons for attrition were not documented. One study [61] had high risk of reporting bias as not all outcomes included in the protocol were reported and three studies received unclear ratings as the protocol was not available [59, 60, 62]. Three studies did not report details of allocation concealment [59, 60, 62] and two studies did not report details of random sequence generation [58, 60], so were rated as unclear in these domains.

Three studies blinded outcome assessors [52, 53, 56] and for one study [59], participants returned outcomes online so these had low risk for detection bias. Four studies did not report details of blinding procedures for outcome assessments so were rated as unclear. No studies reported blinding of participants, however participant blinding would not have been feasible due to the need for participants to know details of study conditions to give informed consent and breaches of ethical conduct may also influence participant outcomes. Therefore, risk of performance bias decisions were based on the potential for knowledge of participants’ study conditions to influence the behaviour of personnel. Of the three studies that documented any blinding procedures, two clearly specified that “single blind” procedures referred to assessor blinding [53, 56]. Personnel were not blinded but since peer facilitators delivered the active intervention and were not involved in delivering TAU, these two studies were judged to have low risk of performance bias. Similarly, the third study reporting blind procedures [52] stated an “assessor-blind” design was used and no blinding of personnel was documented. However, the two active conditions were delivered by separate personnel, peer facilitators and mhealth specialists [52], so the risk of performance bias was judged to be low. The remaining five studies did not provide any information on blinding procedures so were rated as unclear risk for performance bias [58–62].

Only two studies [52, 53] had low overall risk of bias, due to having low risk for all individual domains of bias. Since one study [60] had high risk of attrition bias and one study had high risk of reporting bias [61], these both had high overall risk of bias. The remaining four studies had unclear overall risk of bias, due to having unclear risk of selection bias [58, 59, 62], attrition bias [56] or reporting bias [59, 62].

### Study outcomes and quantitative synthesis

No studies reported outcomes at follow-up exceeding 12 months so outcomes are described at two time-points; post-intervention (end of “treatment”) and follow-up (less than 12 months post-intervention). Six trials [52, 53, 56, 58, 61, 62] provided usable data for meta-analyses, providing data for 1626 participants (76% of all participants). Results of the main analyses are displayed in Table 2. Forest plots for the main analysis, subgroup analysis and sensitivity analysis are displayed in the supplementary material (Additional file 1). No studies reported outcomes related to the meaning or connectedness components of the CHIME framework [42], acute service use, independent living or employment outcomes. Findings from studies which did not provide usable data for meta-analysis and for outcomes where there were insufficient studies to conduct meta-analysis are both reported for each outcome below, in addition to the results from the quantitative synthesis summarised in Table 2.

**Table 2:** Results of the main analysis

Outcome	Number of trials	N	SMD (95% CI), *p*-value	Heterogeneity, I^2^; Chi,^2^ df	Duration of follow-up, post end of treatment
*Post-intervention*					
Recovery [52, 55, 56, 58, 62]	5	1265	0.18 (0.07 to 0.29), *p* = 0.002	I^2^ = 0%; Chi^2^ = 4.01, df = 4	
Hope [53, 56, 58]	3	1029	MD = 0.18 (− 0.34 to 0.69), *p* = 0.50	I^2^ = 0%; Chi^2^ = 1.68, df = 2	
Empowerment [57, 58, 61, 62]	4	750	0.17 (− 0.07 to 0.40), *p* = 0.17	I^2^ = 55%; Chi^2^ = 6.67, df = 3	
Global symptoms [52, 54, 58]	3	823	−0.13 (− 0.27 to 0.01), *p* = 0.07	I^2^ = 0%; Chi^2^ = 1.11, df = 2	
Depression [52, 55, 58, 62]	4	929	−0.09 (− 0.22 to 0.04), *p* = 0.18	I^2^ = 0%; Chi^2^ = 0.99, df = 3	
*Follow-up*					
Recovery [52, 55, 56, 62]	4	983	0.21 (0.08 to 0.34), *p* = 0.002	I^2^ = 5%; Chi^2^ = 3.16, df = 3	3 to 6 months
Empowerment [57, 61, 62]	3	487	0.13 (−0.05 to 0.31), *p* = 0.14	I^2^ = 0%; Chi^2^ = 0.37, df = 2	3 weeks to 6 months
Depression [52, 55, 62]	3	674	−0.12 (− 0.27 to 0.03), *p* = 0.11	I^2^ = 0%; Chi^2^ = 0.95, df = 2	3 to 6 months

Means and Standard Deviations for TAU and clinician-led comparator group were combined for Eisen 2012. *MD* Mean Difference, *SMD* Standardised Mean difference, *CI* confidence interval, *SMDs* are reported unless stated otherwise, *N* number of participants providing outcome data, *df* degrees of freedom

The maximum number of trials included in any meta-analyses was five, so no statistical tests of funnel plot asymmetry were carried out. Planned subgroup analyses of structured and unstructured interventions were not possible as all studies providing usable data for meta-analyses were structured interventions. Subgroup analysis that included only studies solely involving participants with mental health conditions defined as severe was conducted for empowerment at post-intervention by removing the only study providing usable data for meta-analyses without a participant population with severe mental health conditions [61]. For the main analysis of all other outcomes, only studies including participants experiencing severe mental health conditions provided usable data. Planned sensitivity analyses of studies with low overall ROB werenot possible, since only two studies [52, 53] met the criteria for low overall ROB. TAU only sensitivity analyses wereconducted for recovery, hope, empowerment and depression outcomes by removing the study with an active comparator [52] and using only TAU data for the three-armed trial [58].

### Personal recovery outcomes

#### Recovery

Five trials providing post-intervention data which were useable in meta-analyses found strong evidence for a small effect of group peer support on recovery. One study did not provide usable data for meta-analyses and found no evidence for an effect of the intervention on recovery [59]. Sensitivity analyses including only studies with TAU control groups and excluding a study which used an outcome measure that was not fully validated [62] did not differ substantially from the results of the main analysis.

Four trials provided usable follow-up data for meta-analysis, which found strong evidence for a small effect of group peer support on recovery at three- and six-months follow-up. Results of sensitivity analysis including only studies using TAU control groups did not differ substantially from the main analysis.

Of the two studies reporting recovery with low overall ROB, one study reported evidence for an increase in recovery for participants receiving WRAP relative to TAU [55] at both post-intervention and six-month follow-up, and one study reported no evidence for a statistically significant difference in recovery between the two conditions found at either time point [52].

#### Hope

Three trials provided usable post-intervention data for meta-analysis, which found no evidence for an effect of group peer support on hope. Sensitivity analysis using only studies with TAU control groups did not alter this result. Only two studies reported follow-up data for hope, so meta-analyses were not possible. One study reported evidence for an effect [53] and the other reported no effect [56] of group peer support on hopeacross post-intervention and 6 months follow-up.

#### Empowerment

Self-advocacy was reported by two studies [54, 57], which we categorised as an empowerment outcome because it shared concepts with empowerment such as assertiveness and self-direction [66]. One study found evidence for increased self-advocacy following the intervention [54] and the other no effect [57], relative to TAU. Since the same authors used measures of both self-advocacy and empowerment, measures of self-advocacy were excluded from meta-analyses. One study reporting no usable data for meta-analyses found no evidence for an effect of the intervention on empowerment [59]. Four trials provided usable post-intervention data for meta-analysis, which found no evidence for an intervention effect on empowerment. Sensitivity analysis only including studies with TAU control groups and subgroup analysis only including studies with participants experiencing mental health conditions defined as severe did not alter these results.

Three trials reported usable follow-up data formeta-analysis, which found no evidence for an effect of group peer supporton empowerment at 3 weeks, 3 months and 6 months follow-up. No sensitivity or subgroup analyses were conducted as all studies included used a TAU control and only two studies had participant populations with mental health conditions defined as severe.

#### Identity

All three anti-stigma intervention trials reported self-stigma [60–62], which we categorised within the domain of recovery. Trials reported data at post-intervention and at follow-up of 3 weeks [61], one month [60] or 3 months [62]. Interventions effects on identity were mixed, with one study reporting evidence for a reduction in self-stigma relative to TAU [62] and one study reporting no difference between groups [61] across the full study periods. One study reported improvements relative to TAU for two subscales and no effect for two subscales of a self-stigma measure at both time points [60]. This study did not provide useable data, so identity could not be quantitively synthesised.

#### Quality of life

Three studies reported quality of life at post-intervention [52, 53, 59] and two studies reported follow-up at three months [52] or 6 months [53]. Evidence for intervention effectiveness was mixed with one study reporting evidence for improvements in quality of life relative to TAU across the full study period [53] and two studies reported no difference [52, 59], with one of these two studies [59] providing no usable data, so this outcome could not be quantitively synthesised.

#### Self-efficacy

Two studies reported self-efficacy at post-intervention and at follow-up of 3 weeks and 3 months respectively and found no evidence for an effect of the intervention [61, 62].

### Clinical recovery

#### Psychiatric symptoms

One study reported anxiety [55], with evidence for improvements following the intervention relative to TAU across post-intervention and six-month follow-up. Another study reported psychosis [52] and found no difference between groups at either post-intervention or three-month follow-up. Since some studies included both global symptom severity and depression outcomes, these were analysed separately.

##### Global symptoms

One study reporting global symptoms found no evidence for an effect of the intervention relative to TAU but provided no usable data for meta-analyses [59]. In post-hoc analysis, this study reported weak evidence that participants with high use of the online intervention experienced more symptoms than those with low or no use at post-intervention, and an increase in symptoms between four and 12 months [59]. However, the direction of the relationship for causal inference could not be established [59]. Threetrials provided usable post-intervention data for meta-analysis, whichfound weak evidence for an intervention effect in the direction of symptom reduction, though the magnitude of this effect was found to be negligible. Planned sensitivity analyses were not possible due to an insufficient number of studies.

Since only two trials reported follow-up data [52, 53] meta-analyses were not possible. One study reported evidence for reductions in symptoms following the intervention relative to TAU across time [53] and the other reported no between-group differences at 3 months follow-up [52].

##### Depression

One study [60] providing no usable data for meta-analyses reported evidence for a reduction in depressive symptoms following group peer support relative to TAU for women but not for men at post-intervention. Four trials provided usable post-intervention data for meta-analyses, which found no evidence for an effect of group peer support on depression. Sensitivity analyses including only studies with TAU control groups did not alter this result. Three trials provided usable follow-up data for meta-analysis, which found no evidence for an effect of group peer support on depression at three- and six-monthsfollow-up. Sensitivity analyses were not possible due to an insufficient number of studies.

### Social outcomes

#### Social support

One study [59] reported social support at post-intervention and found no evidence for an effect of the intervention. No further studies reported social support or any other social outcome.

## Discussion

### Summary of findings

This review represents a synthesis of findings from trials of group peer support. All studies included in the meta-analyses were structured peer support groups. We found evidence that group peer support may make small improvements to overall personal recovery for people with mental health conditions that are maintained at follow-up of up to 6 months. This effect was unaltered by sensitivity analyses. However, we found no evidence for an effect on empowerment, hope or depressive symptoms either after the intervention or at follow-up. There was weak evidence that group peer support may influence psychiatric symptoms following the intervention but the size of effect for improvement was negligible. These findings cannot offer conclusive evidence for the effectiveness of group peer support for clinical and recovery outcomes, as it was not possible to solely analyse studies with a low overall risk of bias, due to an insufficient number of studies meeting these criteria for planned sensitivity analyses. Quantitative syntheses of most outcomes included in our review protocol were not possible due to none or only one to two of the included studies reporting them. Only one included study was a mutual support intervention, which did not report evidence for an effect on any outcome and had an unclear overall risk of bias on the findings. The study also found evidence of an association between greater use of the online intervention and more difficult experiences of psychiatric symptoms, although the direction of effect was unclear. This study included a measure of social support and was the only study reporting any outcome from the social outcomes group. There was mixed descriptive evidence on the impact of anti-stigma interventions on identity and for self-management interventions on quality of life. Anti-stigma intervention studies reported no descriptive evidence for an effect on self-efficacy, though these similarly had some considerable risks of bias on findings.

### Strengths and limitations

To our knowledge, this is the first review to focus solely on evidence for the effectiveness of group peer support interventions, delivered only by people with lived experience of mental health conditions. This reduced heterogeneity in methods of intervention delivery and statistical heterogeneity was low for the meta-analyses, suggesting relative consistency in intervention effects across studies [67]. There was a distinction in focus between interventions that aimed to reduce self-stigma [10] and those that aimed to improve self-management. Effectiveness for improving recovery may differ between intervention subtypes, however only one included anti-stigma intervention reported recovery [62], so it was not possible to analyse these separately. Variation in participant characteristics was a source of clinical heterogeneitybetween studies [68]. Main analyses of all outcomes except empowerment, however included only participants experiencing mental health conditions that were defined as severe, providing a specific evaluation of intervention effectiveness for these outcomes for people with these experiences. Full appraisal of the effectiveness of group peer support for people with other mental health conditions was not possible due to current limitations of the evidence-base.

Since the focus of this review was intervention effectiveness, we included only RCTs to enhance the potential for causal inference and reduce the influence of bias on the findings [69]. Conversely, this may have limited the studies returned by the search and therefore, the scope of the meta-analyses. We also excluded cluster RCTs since we characterized group peer support as a discrete intervention, which can be randomised at the individual level. However, many mutual support and peer support programs have arisen out of user-led organizations [70], which might more parsimoniously function as the unit of randomisation. Of our 12 methodological exclusions, only one of these was due to the study being a cluster RCT [71]. However, the study did not meet other inclusion criteria, for example, the intervention included both group and one-to-one components [72]. Therefore, although it is unlikely that our exclusion of cluster RCTs has altered the findings of this review, future reviews of group peer support may wish to include this study design within inclusion criteria in order to minimise the risk of missing relevant evidence.

We adopted strict and limited eligibility criteria for this review in order to present a comparable group of interventions for which group peer support was the active ingredient, and to enable valid comparisons of intervention effects. However, this approach may have led to relevant evidence being missed, which could provide interesting and important contributions to our current knowledge of group peer support interventions. For example, we excluded all interventions with any one-to-one support elements. This may have led to the exclusion of potentially helpful programs, which blended group and one-to-one approaches. Combined one-to-one and group peer support programs may be particularly beneficial for flexibly accommodating the diverse needs of people using peer support interventions and require evaluation and synthesis in future reviews.

Our adoption of strict eligibility criteria for the review attempted to address the heterogeneity peer support interventions, through focusing on the effectiveness of one narrowly defined sub-type. However, this may limit the generalisability of these findings to other peer support interventions. Only one included study met our definition of mutual support, which was delivered online, so findings may not be generalisable to face-to-face groups due to distinctive barriers to peer support utilisation delivered via technology [73]. Therefore, the review findings are specifically generalisable to structured peer support groups. Studies were predominantly conducted in America, which may further limit the generalisability of the findings identified here. We also adopted a strict definition of peer support to exclude all health professional involvement. However, some group peer support interventions are often co-delivered with health professionals and maintain a non-diagnostic, recovery-orientated ethos, such as peer support groups provided internationally by the Hearing Voices Network [74]. These groups may have many benefits for recovery and require independent evaluation. Similarly, we excluded all groups with any focus other than promoting recovery with mental health conditions. This was to enable us to report any impact on recovery outcomes as direct effect of the interventions, rather than as possible secondary benefits experienced through addressing other issues, such as bereavement or physical health conditions. Peer-led and delivered group interventions targeting experiences commonly experienced by people who experience mental health conditions may also have benefits for recovery. These require independent syntheses and may further contribute to the evidence-base for the effectiveness of group peer support interventions.

Of the 4277 papers returned by our search, only 11 met our eligibility criteria for inclusion, reporting findings of eight trials. However, we used intentionally broad search terms in order to collect a large number of papers and to ensure that no potentially eligible studies were missed (see Supplementary Material, Additional file 1 for full search strategy). A large number of papers were also excluded at the full text screening stage. We were conservative about retrieving full text studies and retained all papers with any evidence of relevancy for detailed consideration. There were some studies that proved problematic for eligibility decisions, included in the supplementary material (Additional file 1). If there was any doubt that a study met eligibility criteria it was excluded, in accordance with recommended procedures for systematic reviews [32].

A methodological limitation of this review was the omission of terms related to “consumer” within the intervention terms of our search strategy, included in Appendix 1 of the Supplementary Material (Additional file 1). In North America, Australia and other countries outside of the UK, this term is often used to describe people who use mental health services. Our initial drafts of our search strategy did include a larger number of terms for peers, including the term “consumer”. However, when piloting our search terms we found that a simplified search, excluding some intervention terms, continued to pick up all our model papers and streamlined the results more closely to our inclusion criteria. In spite of these considerations, we cannot rule out the possibility that our reduced search strategy may have missed some relevant studies. This shortcoming highlights the difficulties of conducting reviews in fields where the language used is not well-defined and varies across study locations.

At the stage of peer review, it was highlighted that the inclusion of social support as an outcome for appraising the effectiveness of group peer support may be problematic, since initiating an intervention involving contact with others may physically increase social support. Only one study included in the present review included social support as an outcome and found no evidence for an effect of the intervention. This issue of circularity is particularly pertinent with respect to studies that do not include follow-up measurements beyond the end of the duration of the intervention. Only one included study reported social support as an outcome, which was assessed during and at the end of the intervention but not at longer-term follow-up. However, the study reported no effect of the intervention on social support. In order to appraise the impact of group peer support interventions on social support, it may be necessary for future studies to consider follow-up points beyond the end of the intervention. If any change in the outcome is maintained, this would be a more reliable indicator of any effect of the intervention.

### Interpretation and contribution to the evidence-base

The findings of this review contribute to the mixed evidence-base for the effectiveness of peer support interventions based on findings from RCTs. Similarly, to the earlier review by Lloyd-Evans and colleagues [21], interventions categorised as peer support services were found to improve recovery but not empowerment. Previous reviews have found that group peer support may increase empowerment [10] and hope [25], however, not all studies included in these reviews met our eligibility criteria, often due to the involvement of non-peer professionals in the delivery or moderation of the intervention. Compared to the more recent review [10], this may have reduced the power of the meta-analyses to detect a small effect across studies. Since empowerment is a component of recovery [42] and the effect of group peer support on recovery is small, intervention effects on recovery components are less likely to be detected by smaller studies and meta-analyses.

The meaning of recovery may differ between different individuals as it is a personally defined process [75] and since peer support is a complex intervention, it may also work in different ways for different individuals. Therefore, individual domains of recovery may change at different rates within the recovery process, though broader measures of recovery are more able to capture overall improvement within the short timeframe of most included RCTs. Although further high-quality studies are needed to fully rule out potential influences of bias on study findings [21], the findings of this review are indicative of a positive effect of group peer support on recovery. Four of the five studies included in the quantitative synthesis were self-management interventions, which suggests this intervention-type may be effective for recovery. It is worth noting that sensitivity analyses using just TAU comparison groups did not alter findings for recovery, though only two studies [52, 58] employed active comparator conditions involving non-peer clinicians, which tentatively suggests that structured peer-delivered self-management interventions may be comparably effective for enhancing recovery to those delivered by other providers. This supports the findings of a previous review [76], which found no difference in the effectiveness of interventions delivered by peer and non-peer providers for improving recovery outcomes. All self-management interventions involved contributions of examples from the lived experience of group facilitators, and recovery-orientated education, suggesting that recovery may be exemplified through practical strategies suggested by facilitators and group members, which could contribute to experiential knowledge and intervention effectiveness [77]. However, it is possible that within peer support interventions delivering a structured curriculum, the potential for the exchange of experiential knowledge developed through individual experience may be limited. Mutual support groups might offer the potential to increase recovery through the sharing of personalised experiential knowledge [15] and coping strategies [78] though the relative absence of these trials in the literature prohibited comparisons of these intervention types on recovery outcomes.

Previous reviews have found no evidence for an effect of group peer support on global symptoms [25] and no difference in symptoms compared to TAU [25], or to non-peer providers [76], across peer support interventions. Interpretation of our findings for global symptoms as fully consistent with those of previous reviews is complicated by the small number of trials contributing to the meta-analysis and heterogeneity in trial design, since one study [52] compared two self-management interventions. This may have reduced the relative effectiveness of group peer support for symptoms since self-management interventions, delivered by either peers or non-peers, were found to improve psychiatric symptoms by a recent review and meta-analysis [79] and the study included in the present review found evidence for improvements within both groups [52]. Previous reviews have also found more consistent evidence for peer-delivered self-management interventions than other forms of peer support [9], though the present review found no evidence for an effect of group peer support on depressive symptoms. It has been suggested that recovery outcomes may be more appropriate than clinical outcomes for assessing the effectiveness of peer support [26], since the aim of interventions are to improve recovery rather than to eliminate symptoms [75], which may still be present throughout the process of reclaiming personal well-being and satisfaction in life [40]. However, it was not possible to assess the impact of group peer support on other outcomes that may be important for recovery, such as quality of life or social outcomes [30], as either no or few studies reported these. These outcomes may also have greater value to many individuals with lived experience of mental health conditions than traditional clinical outcomes [80].

Our findings for group peer support broadly parallel those of the concurrent review by White and colleagues [31] for the effectiveness of one-to-one peer support for improving outcomes for people using mental health services. The available evidence base for one-to-one peer support similarly suggests that interventions may be more likely to improve personal recovery than outcomes related to clinical recovery. Both reviews indicate a small positive effect for recovery, from a similar number of trials, indicating that this may be a consistent effect for peer support, irrespective of whether the intervention is delivered individually or in groups. Although our review does offer a tentative suggestion for a potential intervention effect on global symptoms, which could later be confirmed through expansions to the evidence-base, our more positive finding may be explained by the high representation of self-management interventions in the synthesis [9] rather than by the format of delivery. In the case of both reviews, the use of lived experience within included interventions in relation to its hypothesised contribution to the mechanisms of effect is rarely described, which could be further specified in order to fully appraise the mechanisms of peer support. Comparably to the findings of the present review, White and colleagues also note the limited number of studies reporting each outcome and the continued presence of some risks of bias to included study findings, limiting interpretation of the available evidence base for both approaches and its utility for informing policy and service developments.

### Research implications

The findings of this review highlight the current paucity of evidence from high quality trials of group support interventions needed to draw firm conclusions about effectiveness for a broad range of outcomes. As a result, many reviews of peer support have combined heterogeneous groups of interventions to attempt to appraise effectiveness [26, 78]. The present findings suggest one distinction in terms of anti-stigma and self-management as subcategorizations within existing typologies, based on a limited number of included studies. The question of the most effective forms of peer support within different settings remains [26] and cannot fully be addressed by meta-analytic approaches at present, due to an insufficient number of trials to group interventions appropriately [78]. Future trials could clearly define the model of group peer support used and ensure people with mental health conditions adopt leadership roles in the design of the intervention, to ensure lived experience expertise is optimised [81].

A more holistic appraisal of effectiveness for recovery would also be facilitated by the inclusion of a broader range of outcomes and service settings in order to expand the current evidence-base. In particular, there is a current lack of high-quality trials of mutual support group interventions, in spite of the high prevalence and uptake of this form of mental health support across the UK and the United States [82] and the large body of qualitative literature detailing personal benefits derived through this form of intervention [20]. Trials of group peer support interventions to improve outcomes for people diagnosed with common mental health conditions are virtually absent in the literature and these are also strongly encouraged. Expansions to the current evidence-base could establish more conclusive evidence for a positive effect of group peer support on recovery outcomes. Future reviews could then determine the specific effectiveness of structured and unstructured interventions, self-management and anti-stigma interventions, and for different clinical groups, to guide implementation within primary and secondary care settings. The present review found no evidence that small improvements in recovery were due to changes in hope or empowerment. Although these findings were based on a limited number of studies, this raises questions regarding causal mechanisms of existing group interventions. It is possible that increases in recovery could be caused by changes in component processes such as meaning or connectedness [42], which were not reported by included studies and future studies could include measurements of these. Qualitative accounts of individuals participating in group peer support interventions, both as process evaluations embedded within trials and as independent studies could indicate the elements of the intervention that are helpful and mechanisms of effect [83]. This may be particularly informative for determining whether self-management is an essential intervention component for improving recovery. Previous reviews [11, 12, 84] have provided useful summaries of proposed mechanisms of effect for peer support interventions, which have also been identified in qualitative analysis [18]. Future group peer support interventions need a clear theory of change and proposed mechanism of hypothesised effect as it is uncertain how any of the positive results presented were achieved from the included studies.

### Policy and practice implications

The findings of this review and of other reviews that have included group peer support approaches [8, 10, 25, 26] are promising with respect to the potential for group peer interventions to enhance recovery for people using mental health services. The current evidence base, however comprises a small number of trials of heterogeneous group interventions, often with considerable risks of bias to study findings. There is also limited available evidence to make conclusions about effectiveness for a broad range of outcomes that may be important for recovery, particularly social outcomes. This prohibits recommendations for the routine implementation of specific forms of group peer support across mainstream services at present. Some negative psychological outcomes have been reported previously by a trial of an online mutual support intervention for women with breast cancer [85] and by a study included in this review [59], in spite of high user satisfaction in both instances. If online mutual support group interventions are adopted by services, these may benefit from moderation, either by peer or non-peer professionals [59], to guard against any potentially negative effects.

The findings of the present and previous reviews [10, 25] suggest that where structured peer support groups are implemented locally, these may make small improvements to personal recovery for individuals accessing these services. International goals to implement recovery-orientated services within mental health systems [86] may also be assisted by increasing implementation of interventionsdelivered by people with lived mental health conditions, ensuring individuals who use mental health services have had a lead role in the development of these [81] in order to truly facilitate the integration of recovery principles and values [87] and cultural change in working practices.

## Conclusion

We found that participation in structured peer support groups may make small contributions to supporting personal recovery for people with lived experience of mental health conditions. Evidence from the few trials available indicated a limited impact on other outcomes. However, we adopted a more limited conceptualisation of group peer support interventions than some previous reviews, which may restrict the generalisability of our findings. All findings should be treated with caution, due to the quality and quantity of available evidence, which is insufficient to make firm policy and practice recommendations at present. Appraisals of intervention effectiveness for many outcomes that may promote personal recovery were not possible due to a near absence from the literature. Group peer support represents a heterogeneous group of interventions: we propose a distinction between anti-stigma and self-management programmes. This review stresses the need for more high-quality trials of group peer support, which consider a broader range of recovery-orientated outcomes, target particular service settings and optimise the use of experiential expertise within both intervention development and delivery.

## Supplementary Information

**Additional file 1.**

**Additional file 2.**

**Additional file 3.**

## Data Availability

All data generated or analysed during this study are included in this published article and its additional files.

## Abbreviations

RCT: Randomised Controlled Trial
PRISMA: Preferred Reporting Items for Systematic Reviews and Met-Analysis
CENTRAL: Cochrane Central Register of Controlled Trials
PRESS: Peer Review of Electronic Search Strategies
TAU: Treatment As Usual
CHIME: Connectedness Hope Identity Meaning Empowerment
ROB: Risk of Bias
RevMan: Review Manager
SMD: Standardised Mean Difference
WRAP: Wellness Recovery Action Planning
WLC: Waiting List Control
BRIDGES: Building Recovery of Individual Dreams and Goals
COP: Coming Out Proud
