# Supplementary material for: A systematic review and meta-analysis of group peer support interventions for people experiencing mental health conditions
Source: BMC Psychiatry. 2021 Jun 23;21:315. doi: 10.1186/s12888-021-03321-z (PMC8220835; doi:10.1186/s12888-021-03321-z)
Supplement: Supplementary file 1 — Additional file 1. [file 12888_2021_3321_MOESM1_ESM.docx]

Supplementary Material

Appendix 1: Search Strategy

Search Narrative

Database: Ovid MEDLINE(R) and Epub Ahead of Print, In-Process & Other Non-Indexed Citations and Daily

2019>

Host: OVID

Search parameters: 1946 to July 12, 2019

Date of search: Saturday July 13^th^ 2019

Conceptual narrative: The purpose of this literature search was to identify studies reporting randomised controlled trials of peer support interventions delivered in a group setting. The population requirement of the review – that the population are adults with an existing mental health condition – will be operationalised at screening.

| Search strategy | Contextual narrative |
| --- | --- |
| 1 (Peer* adj3 (support* or led or lead* or deliver* or run* or held or direct* or online or "on line" or  forum*)).ti,ab,kw. (7795)  2 ("peer-run" or "peer-led").ti,ab,kw. (920)  3 ((lay or layperson* or laypeople or "lay people") adj3 (support* or led or lead* or deliver* or run* or held or  direct* or online or "on line" or forum*)).ti,ab,kw. (855)  4 (Paraprofessional* adj3 (support* or led or lead* or deliver* or run* or held or direct* or online or "on line" or  forum*)).ti,ab,kw. (86)  5 (mutual* adj2 (aid* or support* or help*)).ti,ab,kw. (1764)  6 1 or 2 or 3 or 4 or 5 (10386) | Lines 1-5 set out the intervention terms of the literature search.  Lines 1, 2, 3 & 5 use proximity searching, indicated by adj3. This means that the term peer* will be searched within two words of the term support*, allowing for other words which may appear between these two words (such as leader e.g. Peer leaders support) in the title or abstract of a study. Proximity searching also allows for the terms to appear in any order, such that a study that reported ‘supporting leadership through peers’ would also be identified.  Phrase searching is used at line 2. Here the use of speech markers means that only the phrase “peer-led” will be searched for. This search line was introduced as line 1 did not identify a relevant study by van Gestel-Timmermans because of the hyphen in ‘peer-led’.  All the searching is undertaken on the title (ti), abstract (ab) and author generated keyword (kw) fields.  Line 6 combines all of the intervention search terms using the Boolean connector OR. This means that all f the search terms set out in lines 1-5 will be searched for. |
| 7 Group*.af. (4098399) | The research question aims to address the effectiveness of peer support interventions when delivered in a group setting. The purpose of line 7 is to introduce the setting context to the literature search.  The search uses truncation (indicated by *) so that group or groups are searched for. The search is executed on all fields (indicated by .af.) which includes title, abstract or keyword and also indexing terms. This includes the indexing term peer group. |
| 8 randomized controlled trial.pt. (485396)  9 controlled clinical trial.pt. (93156)  10 (randomized or randomised).ab. (536692)  11 placebo.ab. (199175)  12 clinical trials as topic.sh. (187637)  13 randomly.ab. (314437)  14 trial.ti. (201606)  15 8 or 9 or 10 or 11 or 12 or 13 or 14 (1260058)  16 exp animals/ not humans.sh. (4598182)  17 15 not 16 (1160492) | Lines 8-17 are a study design literature search filter to identify studies reporting RCT or CCT. The search filter used here is the Cochrane HSSS sensitivity and precision maximising version.^1^ |
| 18 6 and 7 and 17 (1298) | Line 18 concludes the literature search in MEDLINE by combining search terms for: interventions (line 6) AND setting (line 7) AND study design (line 17). This focuses the search on studies which report all three components of the search strategy in the title or abstract. |

Search results by database:

PsychINFO

Database name: PsychINFO

Host: OVID

Data parameters: 1806 to July week 2 2019

Date searched: 13.07.2019

Search strategy:

Database(s): PsycINFO 1806 to July Week 2 2019
Search Strategy:

| **#** | **Searches** | **Results** |
| --- | --- | --- |
| 1 | (Peer* adj3 (support* or led or lead* or deliver* or run* or held or direct* or online or “on line” or forum*)).ti,ab. | 9331 |
| 2 | (“peer-run” or “peer-led”).ti,ab. | 758 |
| 3 | ((lay or layperson* or laypeople or “lay people”) adj3 (support* or led or lead* or deliver* or run* or held or direct* or online or “on line” or forum*)).ti,ab. | 609 |
| 4 | (Paraprofessional* adj3 (support* or led or lead* or deliver* or run* or held or direct* or online or “on line” or forum*)).ti,ab. | 223 |
| 5 | (mutual* adj2 (aid* or support* or help*)).ti,ab. | 2524 |
| 6 | 1 or 2 or 3 or 4 or 5 | 12537 |
| 7 | Group*.af. | 1483387 |
| 8 | Random*.af. | 533076 |
| 9 | (“Phase 3” or “phase3” or “phase III” or P3 or “PIII”).ti,ab. | 6906 |
| 10 | 8 or 9 | 537988 |
| 11 | 6 and 7 and 10 | 1987 |

Embase

Database name: Embase

Host: OVID

Data parameters: 1974 to July 12^th^ 2019

Date searched: 13.07.2019

Search strategy:

Database(s): Embase 1974 to 2019 July 12
Search Strategy:

| **#** | **Searches** | **Results** |
| --- | --- | --- |
| 1 | (Peer* adj3 (support* or led or lead* or deliver* or run* or held or direct* or online or "on line" or forum*)).ti,ab,kw. | 10635 |
| 2 | ("peer-run" or "peer-led").ti,ab,kw. | 1222 |
| 3 | ((lay or layperson* or laypeople or "lay people") adj3 (support* or led or lead* or deliver* or run* or held or direct* or online or "on line" or forum*)).ti,ab,kw. | 1048 |
| 4 | (Paraprofessional* adj3 (support* or led or lead* or deliver* or run* or held or direct* or online or "on line" or forum*)).ti,ab,kw. | 106 |
| 5 | (mutual* adj2 (aid* or support* or help*)).ti,ab,kw. | 2276 |
| 6 | 1 or 2 or 3 or 4 or 5 | 13895 |
| 7 | Group*.af. | 6286362 |
| 8 | Random*.af. | 1643919 |
| 9 | ("Phase 3" or "phase3" or "phase III" or P3 or "PIII").ti,ab,kw. | 111042 |
| 10 | 8 or 9 | 1706868 |
| 11 | 6 and 7 and 10 | 1767 |

Cochrane (CENTRAL)

Database name: Cochrane Central Register of Controlled Trials

Host: Cochrane Library

Data parameters: Issue 7 of 12, July 2019

Date searched: 13.07.2019

Search strategy:

Search Name: Cochrane peer support search

Date Run: 13/07/2019 13:49:27

Comment: Cochrane (CENTRAL) peer support search

ID Search Hits

#1 ((Peer* NEAR/3 (support* or led or lead* or deliver* or run* or held or direct* or online or "on line" or forum*))):ti,ab,kw OR (("peer-run" or "peer-led")):ti,ab,kw OR (((lay or layperson* or laypeople or "lay people") NEAR/3 (support* or led or lead* or deliver* or run* or held or direct* or online or "on line" or forum*))):ti,ab,kw OR ((Paraprofessional* NEAR/3 (support* or led or lead* or deliver* or run* or held or direct* or online or "on line" or forum*))):ti,ab,kw OR (mutual* NEAR/2 (aid* or support* or help*)):ti,ab,kw 2481

#2 (Group*) 1127442

#3 #1 AND #2 2146

(of these 2101 were “trials” in the trials tab):

**2101 Trials matching "#3 - #1 AND #2"**

[*Cochrane* Central Register of Controlled Trials](https://www.cochranelibrary.com/)

Issue 7 of 12, July 2019

Appendix 2: Forest Plots

Main analyses

Personal Recovery Outcomes:

1)a. Recovery at post-intervention


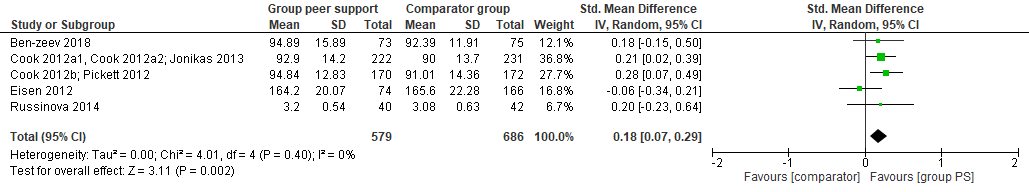


1)b. Recovery at follow-up


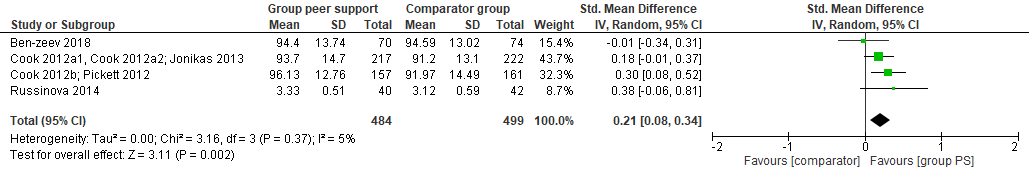


2) Hope at post-intervention


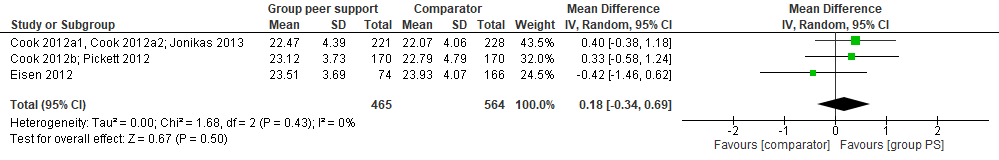


3)a. Empowerment at post-intervention


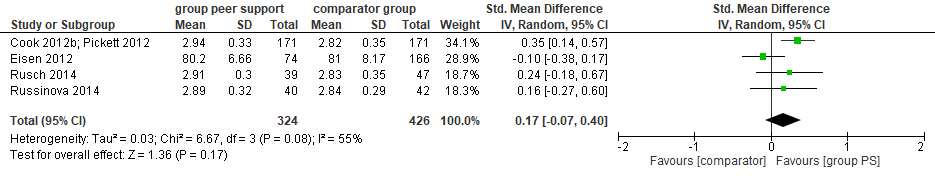


b) Empowerment at follow-up


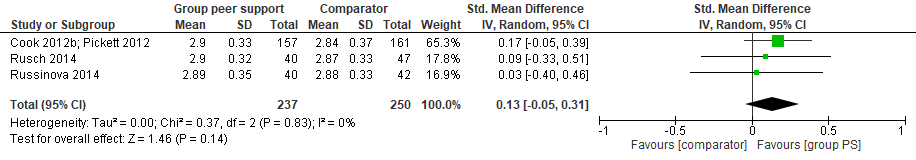


Clinical Recovery Outcomes:

1) Global Symptoms at post-intervention


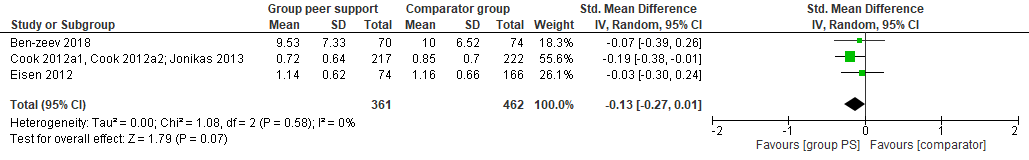


2)a. Depression at post-intervention


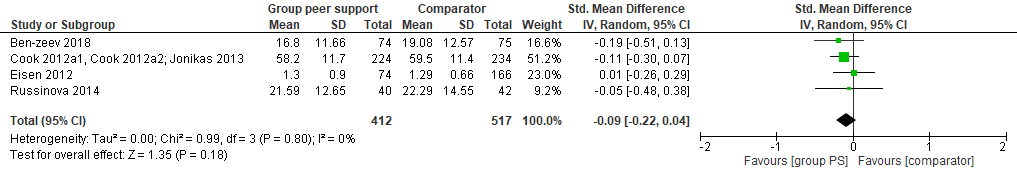


2)b. Depression at follow-up


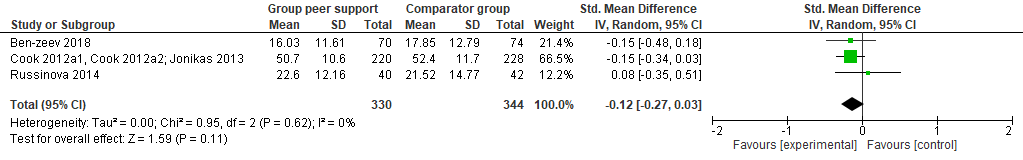


Sensitivity Analyses:

Leave one out meta-analysis for recovery at post-intervention


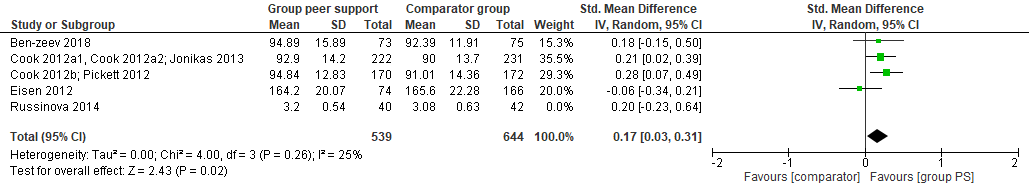


Treatment as Usual Sensitivity analyses

1. a) Recovery post-intervention:


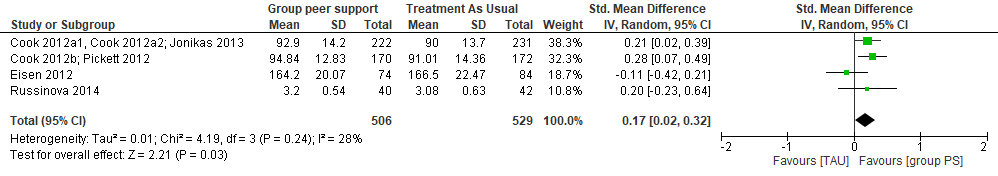


1.b) Recovery at follow-up


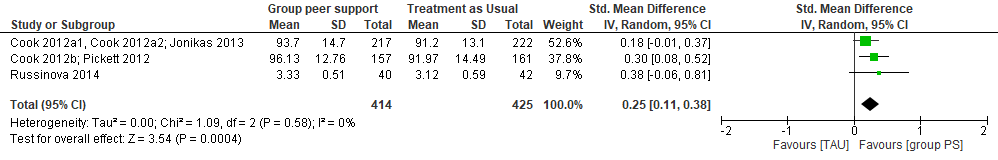


2. Hope at post-intervention


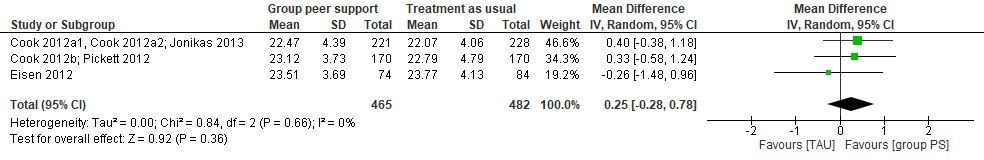


3. Empowerment at post-intervention


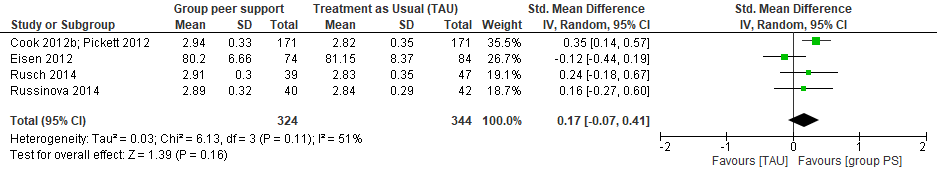


4. Depression at post-intervention


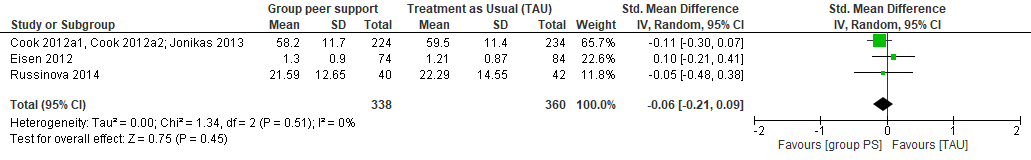


Subgroup analysis

Studies providing usable data for meta-analyses above for hope, recovery, psychiatric symptoms and depression all solely included participants with severe mental health conditions, therefore subgroup analysis was only possible for Empowerment. Five study populations were categorised as solely including people with mental health conditions defined as severe due to inclusion of functional impairment in inclusion criteria (Cook 2012a; Cook 2012b) or secondary mental health service use within a hospital (Eisen 2012), psychiatric rehabilitation service (Russinova 2014) and agency provider of services for people with mental health conditions defined as severe (Ben-Zeev 2018). One study providing usable data for meta-analyses of empowerment at post-intervention (Rüsch 2014) included participants with mixed mental health conditions so has been removed from the subgroup analysis below. This did not change the results for empowerment (no evidence for an effect of the intervention).

Empowerment at post-intervention: people experiencing severe mental health conditions


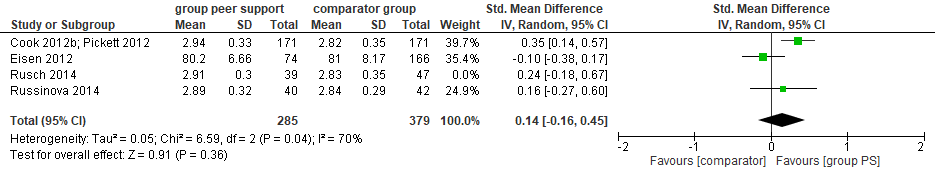


Appendix 3: Problematic eligibility decisions

| Study ID | Eligibility decision | Problematic element of eligibility decision |
| --- | --- | --- |
| Mathews 2018^2^ | Exclude | Limited one to one support:  This intervention involved peer facilitators checking-in with participants in-between sessions. Although the one-to-one support provided was minimal, we defined the intervention as complex and continued with our blanket decision to exclude all interventions with any support provided outside the group. |
| Field 2013^3^ | Exclude | Limited involvement of Health professionals:  This study involved a health professional who was present in the comparator condition. However, the health professional was described as remaining “silent” throughout the group. We excluded this study as we felt that it would be impossible to ascertain the effects of the presence of a health professional on the group and could not be certain whether the professional could have remained silent in every session. This enabled us to maintain our blanket decision to exclude all interventions with any level of health professional involvement in the group, no matter how minimal. |
| [Rüsch](https://www.ncbi.nlm.nih.gov/pubmed/?term=R%C3%BCsch%20N%5BAuthor%5D&cauthor=true&cauthor_uid=24434073) 2014^4^ | Include | Uncertainty regarding facilitator lived experience  The paper reported that two peers delivered the intervention to all groups “except one”. We weren’t sure if this meant that one group had been delivered by non-peers. Therefore, we contacted the authors. We included the study as the authors informed us that for one of the groups only one of the two peer facilitators had delivered the group. |
| Corrigan 2015^5^ | Include | Uncertainty regarding participant eligibility  The study did not ask participants about diagnoses and only asked if they identified as experiencing mental health conditions. However, if all participants were recruited from mental health services this would have meant the study was eligible for inclusion. It was not fully clear where the participants were recruited from as the study was advertised in a variety of service settings. We contacted the authors to ask if all services that participants had been recruited from were exclusively for people with mental health conditions and they confirmed that this criterion was met, so we included the study. |
| Eillis 2011^6^ | Exclude | Uncertainty regarding participant eligibility  The study population was student participants experiencing psychological distress. However, the inclusion criteria were students experiencing low to moderate psychological distress and the screening measure used – the Kessler Psychological Distress Scale (K10) ^7^ is used to screen for possible anxiety and depression. Therefore, it was felt that participants reaching low and moderate thresholds may have mental health conditions. However, through scoping the literature about the K10 we found that participants scoring under 30 are unlikely to have mental health conditions ^8^ an participants scoring over 30 in the present study were excluded. Therefore, we excluded the study. |
| Bright 1999^9^ | Exclude | Uncertainty regarding facilitator lived experience  Paraprofessionals in this study facilitated both a mutual support group and a cognitive behavioural therapy group, with comparator groups led by clinicians. The paper noted that all paraprofessionals had previous experience of using self-help organizations so we thought that facilitators were likely to have lived experience of mental health conditions. However, we contacted the authors for confirmation and they informed us that it was not a requirement for paraprofessionals to have lived experience of mental health conditions so the study was excluded. |
| van Gestel-  Timmermans 2012^10^ | Exclude | Uncertainty regarding participant eligibility  The participant population was people with severe mental health conditions. However, the participants self-reported their diagnoses and this was not further confirmed. We felt that if all participants were recruited from mental health services then this would still meet our participant inclusion criteria so we contacted the authors for clarification. The authors informed us that participants had been recruited from multiple sources, including internet advertisements and not all were using mental health services at the time of the study. Therefore, we excluded the study. |

Appendix 4: Further details of included studies

Table 3 Characteristics of Study interventions

| Study | Intervention classification | Intervention Name and Description | Format of delivery | Training of providers | Degree of co-production of intervention | Number of sessions | Duration of sessions | Comparator/  Control |
| --- | --- | --- | --- | --- | --- | --- | --- | --- |
| Ben-Zeev 2018^11^ | Peer Support: self-management (control condition) | Wellness Recovery Action Planning (WRAP):  As described below | Manaulised; Classes delivered by 2 peer facilitators. Examples given from facilitator lived experience. | Advanced facilitator training from specialist WRAP training centre | As below | 12 classes; once weekly | 90 minutes (1.5 hours) | FOCUS (active condition): self-management intervention delivered via smartphone. Components: 1) daily prompts for self-management  2) Clinician dashboard to see participant actions  3) Access to mobile health specialist |
| Cook 2012a^12,13,14^ | Peer support: Self-management | WRAP:  Intervention to develop a personalised plan to maintain wellness. Delivered in classes with structured topics. Topics include: developing wellness strategies, daily maintenance planning, identification of crisis warning signs and crisis planning. | Manualised; Classes delivered by 2 peer facilitators. Examples given from facilitator lived experience. Group discussions, optional homework. | Certificate to teach Wrap from specialist WRAP training centre.  Additional 2.5 days training from researchers | Developed by user of mental health services (M.E. Copeland) in collaboration with a group of peers, professionals and supporters.^15^ | 8 classes; once weekly | 2.5 hours | WLC*:  Participants told they could receive WRAP after study. TAU** for duration of study included: case management, medication, therapy, substance misuse treatment, peer-led support groups residential and employment services. Also accessed by intervention participants. |
| Cook 2012b^16,17^ | Peer Support: Self-management | Building Recovery of Individual Dreams and Goals (BRIDGES):  Educational course about mental health conditions and management. Delivered in classes with structured topics. Topics include: communication and problem-solving skills, recovery, diagnoses and medication, crisis planning. | Classes delivered by 2 peer facilitators. Examples given from facilitator lived experience. Group discussions. | Completed BRIDGES training from specialist BRIDGES training centre. Additional 2.5 days training from researchers. | Curriculum was jointly written by people with severe mental health conditions and professionals from a Tennesse self-help organization and state department. | 8 classes; once weekly | 2.5 hours | WLC*:  Participants told they could receive BRIDGES after study. TAU** for duration of study included: case management, medication, therapy, substance misuse treatment, peer-led support groups residential and employment services. Also accessed by intervention participants. |
| Eisen 2012^18^ | Peer support: Self-management | Vet-to-Vet:  Group sessions structured around set topics and published recovery materials. Example: The Recovery Workbook.^19^  Class topics include: disability awareness and recovery. | Classes delivered by 2 peer facilitators. Examples given from facilitator lived experience. Group discussions.  Use of published recovery materials. | Facilitators received training in delivering vet-to-vet from researchers (Duration not reported) | Developed by a veteran with experience of schizophrenia (M. Armstrong) and mental health professionals at a Veterans Administration Community mental health centre. | 12 classes; once weekly | 45 minutes | 1.TAU**: inpatient services provided at Veterans Administration hospital  2. Clinician-led vet-to-vet: delivered in the same format as peer-led vet-to-vet by a clinician with optional use of recovery materials. |
| Kaplan 2011^20^ | Mutual support | Two unstructured, unmoderated online groups:  1. Listserv – enabled users to send anonymous emails that were received by the whole group.  2. Bulletin-board – users were able to post and read messages on an online board | Unlimited access to online group or listserv | N/A | Researcher developed; no co-production reported | Participants could choose own level of use.  Intervention duration:  12 months | Participants could choose own level of use | WLC*  Participants asked to refrain from using online peer support for duration of study. Option to use intervention after study. |
| Corrigan 2015^5^ | Peer support: Anti-stigma | Coming Out Proud (COP). Intervention to support decisions about disclosure of mental health conditions. Three structured class topics: 1. Costs and benefits of disclosure. 2. Ways of disclosing. 3. Constructing personal stories in disclosure. | Manualised;  Structured group classes delivered by 1 to 2 peer facilitators. Participant workbook | Two 8-hour day training sessions in COP. Facilitators had to exceed 75% on COP fidelity to become a certified trainer. | Developed by researcher, led by a steering-committee of people with mental health conditions. | 3 classes; once weekly or one day long session | 2 hours | WLC*  No details recorded |
| Rüsch 2014^4^ | Peer Support: Anti-stigma | COP as described in Corrigan 2015 above | Manualised; Three structured classes delivered by 2 peer facilitators. Participant workbook. | Training in using COP workbook (duration not specified). Requirement to score 80% on COP fidelity | As described above | 3 classes; once weekly | 2hours | TAU**:  Included medication and counselling or psychotherapy for most (80%) of control participants. |
| Russinova 2014 | Peer Support: Anti-stigma | Anti-stigma Photovoice (ASP): intervention to reduce self-stigma. Components: 1.Photovoice method: taking photos about mental health and stigma and creating narratives around them.  2.Psychoeduation: structured topics including, stigma, stereotypes and stigma coping strategies | Manualised; Classes delivered by 2 to 3 peer-leaders.  Group discussions, use of the camera, experiential exercises to decrease affirmation of stereotypes | Three-day training in ASP curriculum and basic teaching skills. Four of 6 facilitators that delivered the study intervention also had attended photovoice classes. | Researcher developed with extensive input from peer-leaders and users of mental health services. | 10 sessions; once weekly | 90 minutes (1.5hours) | WLC*:  Participants received TAU** for duration of study. All participants were users of the Psychosocial Rehabilitation centre where the study was based and had access to services such as recovery courses, in addition to treatment from usual treatment providers (not recorded). |

*WLC = waiting list control

Table 4 Outcomes and results of individual studies

| Study | Outcomes and measures | Follow-up and attrition | number of intervention sessions attended | Summary of findings |
| --- | --- | --- | --- | --- |
| Ben-Zeev 2018^11^ | 1.Global symptoms;  Symptom Checklist-9 (SCL-9)^21^  2.Depression; Beck-Depression Inventory-II (BDI-II)^22^  3.Recovery; Recovery Assessment Scale (RAS), 24-items ^23^  4.Quality of life (QoL) (author N/R**) | Post-intervention;  3 months follow-up (6 months from baseline)  Number of participants providing data at last follow-up (attrition):  74/82 (10%) FOCUS participants  70/81 (14%) WRAP participants | WRAP:  First week: 48%  (N=39)  3^rd^ week: 42% (N=34)  6ht week: 36%  (N=29)  9^th^ week: 28% (N=23)  FOCUS: mean use  1^st^ week: 5.4 days; 3^rd^ week 4.6 days; 6^th^ week 4.3 days; 9^th^ week 3.9 days | No differences in effectiveness between mobile phone delivered self-management (active condition) and peer support group for any outcome  Within group improvements: at post-intervention and 3 months for both groups for SCL-9 and BDI-II; at 3 months for both group for RAS but for WRAP only at 6 months; at post-intervention for QoL for FOCUS group only |
| Cook 2012a^12,14,13^ | 1.Global psychiatric symptoms; Brief Symptom Inventory (BSI)^24^  2.Anxiety & depression; BSI^24^  3.Hope; Hope Scale^25^  4.Empowerment; Patient Self-Advocacy Scale (PSAS)^26^  5. Quality of life: The World Health Organization's WHOQOL-BREF quality of life assessment^27^  6. Recovery: RAS, 41-items^28^ | Post-intervention;  6 months follow-up (8 months from baseline)  Number of Participants at last follow-up (attrition)  WRAP: 251/276 (4%)  Control: 268/279 (9%) | Average attendance: 5 of 8 classes (mean = 5.05, SD = 3.08). | Improvements across all outcomes for peer support group relative to control group |
| Cook 2012b^16,29^ | 1.Recovery; RAS, 41-item^28^  2.Hope; Hope Scale^25^  3. Empowerment; Empowerment Scale^30^ & PSAS^26^ | Post-intervention;  6 months follow-up (8 months from baseline)  Number of participants at last follow-up (attrition): Intervention: 157/212 (26%)  Control: 163/216 (25%) | Average attendance: 5 of 8 classes (mean = 4.85, SD = 3.34) either in person or by makeup session (over the telephone) | Improvements in recovery and empowerment for peer support group relative to control; no between-group differences for hope or self-advocacy |
| Eisen 2012^18^ | 1.Recovery: RAS, 41-item^28^  2.Hope: Hope Scale^25^  3.Empowerment: Empowerment Scale^30^  4. Social functioning – Veterans short form, mental health component^31^  5. Mental health symptoms: Behaviour & Symptom Identification scale (BASIS-24)^32^ | Post-intervention only  Number of participants at post-intervention (attrition):  Across groups: 240/298 (19%) Numbers at final follow-up: peer-led intervention: 74, Clinician-led intervention: 82, TAU: 84 | Calculated across peer- and clinician-led groups: Participants attended 3.8 groups on average during the 12-week study period, and 26% did not attend any groups | No evidence for improvements in any outcome for peer support relative to either two comparator groups. |
| Kaplan 2011^20^ | 1.Recovery: RAS^28^ – used 20 items.  2.Quality of Life: Quality of life interview^33^  3.Empowerment: Empowerment Scale^30^  4.Global psychiatric symptoms: Hopkins Symptoms Checklist^34^  5. Social support: the Medical Outcomes Study (MOS) social support survey^35^ | 4 months into intervention; post-intervention  Number of participants at post-intervention (attrition):  Listserv: 83/101 (18%)  Bulletin board: 88/99 (11%)  TAU: 88/100 (12%) | Listserv: Monthly numbers of messages ranged from 783 at start to 0 towards end of study.  Bulletin Board: 1/3rd of participants never logged-in. 2141 posts made across participants and full study period. | No improvements for peer support group relative to control for any outcome. |
| Corrigan 2015^5^ | 1. Depression: 10-item Centre for Epidemiological Studies Depression Scale (CES-D)^36^  2. Identity: Self-Stigma of Mental Illness Scale (SSMIS)^37^ | Post-intervention;  1-month follow-up (after completing last COP session)  Number of participants at follow-up (attrition):  COP: 51/107 (52%)  Control: 75/98 (23%) | N/R* | Reductions in late stage of self-stigma for peer support group relative to control but not earlier stages. Depression reported as an interaction with gender: improvements for women following COP but not for men. |
| Rüsch 2014^4^ | 1. Identity: Internalised Stigma of Mental Illness Inventory^38^  2. Empowerment; Empowerment Scale^30^  3. Self-efficacy; 1 item of Stigma Stress Scale^39^ | Post-intervention;  3 weeks follow-up (6 weeks from baseline)  Number of participants at last follow-up (attrition):  COP: 40/50 (20%)  Control: 47/50 (6%) | 40 out of 50 COP participants completed all three sessions. | No improvements for peer support group relative to control group for any of the three outcomes |
| Russinova 2014^40^ | 1.Identity; Internalised Stigma of Mental Illness Inventory^38^  2. Depression; 20 item CES-D^41^  3.Empowerment; Empowerment Scale^30^  4.Self-Efficacy; Generalized Perceived Self-Efficacy Scale^42^  5.Recovery; Personal Growth and recovery Scale (developed by the authors for the study) | Post-intervention; 3 months follow-up  Number of participants at last follow-up (attrition):  Reported overall – 78/82 (5%) | Mean (SD) = 6.93 classes (2.65). 75% of participants (N=30) attended six or more classes | Increase in recovery and reduction in self-stigma relative to control; no between-group differences for other outcomes |

*N/R = Not recorded, TAU = treatment as usual

References:

1. Lefebvre C, Manheimer E, Glanville J. Chapter 6: Searching for studies. In: Higgins JP, Green S, editors. Cochrane Handbook for Systematic Reviews of Interventions Version 510 [updated March 2011] [Internet]. Cochrane Collaboration; 2011. Available from: http://handbook.cochrane.org/

2. Mathews CA, Mackin RS, Chou C-Y, Uhm SY, Bain LD, Stark SJ, et al. Randomised clinical trial of community-based peer-led and psychologist-led group treatment for hoarding disorder. BJPsych Open. 2018;4(4):285–93.

3. Field T, Diego M, Delgado J, Medina L. Peer support and interpersonal psychotherapy groups experienced decreased prenatal depression, anxiety and cortisol. Early Hum Dev. 2013;89(9):621–4.

4. Rüsch N, Abbruzzese E, Hagedorn E, Hartenhauer D, Kaufmann I, Curschellas J, et al. Efficacy of coming out proud to reduce stigma’s impact among people with mental illness: Pilot randomised controlled trial. Br J Psychiatry. 2014;204(5):391–7.

5. Corrigan PW, Larson JE, Michaels PJ, Buchholz BA, Rossi R Del, Fontecchio MJ, et al. Diminishing the self-stigma of mental illness by coming out proud. Psychiatry Res. 2015;229(1–2):148–54.

6. Ellis L, Campbell A, Sethi S, O’Dea B. Comparative randomized trial of an online cognitive-behavioral therapy program and an online support group for depression and anxiety. J cybertherapy Rehabil. 2011;4(4):461–7.

7. Kessler RC, Andrews G, Colpe LJ, Hiripi E, Mroczek DK, Normand SLT, et al. Short screening scales to monitor population prevalences and trends in non-specific psychological distress. Psychol Med. 2002;32(6):959–76.

8. Slade T, Grove R, Burgess P. Kessler Psychological Distress Scale: Normative data from the 2007 Australian National Survey of Mental Health and Wellbeing. Aust N Z J Psychiatry. 2011;45(4):308–16.

9. Bright J, Neimeyer R, Baker K. Professional and Paraprofessional group treatments for depression. A comparison of cognitive-behavioural and mutual support interventions. J Consult Clin Psychol. 1999;67(4):491–501.

10. van Gestel-Timmermans H, Brouwers E, van Assen M, van Nieuwenhuizen C. Effects of a peer-run course on recovery from serious mental illness: a randomized controlled trial. Psychiatr Serv. 2012;63(1):54–60.

11. Ben-Zeev D, Brian RM, Jonathan G, Razzano L, Pashka N, Carpenter-Song E, et al. Mobile health (mHealth) versus clinic-based group intervention for people with serious mental illness: A randomized controlled trial. Psychiatr Serv. 2018;69(9):978–85.

12. Cook JA, Copeland ME, Jonikas JA, Hamilton MM, Razzano LA, Grey DD, et al. Results of a randomized controlled trial of mental illness self-management using wellness recovery action planning. Schizophr Bull. 2012;38(4):881–91.

13. Cook JA, Jonikas JA, Hamilton MM, Goldrick V, Steigman PJ, Grey DD, et al. Impact of Wellness Recovery Action Planning on service utilization and need in a randomized controlled trial. Psychiatr Rehabil J. 2013;36(4):250–7.

14. Cook JA, Copeland ME, Floyd CB, Jonikas JA, Hamilton MM, Razzano L, et al. A randomized controlled trial of effects of wellness recovery action planning on depression, anxiety, and recovery. Psychiatr Serv. 2012;63(6):541–7.

15. Copeland ME. Wellness Recovery Action Plan. Occup Ther Ment Heal. 2002;17(3–4):127–50.

16. Cook JA, Steigman P, Pickett S, Diehl S, Fox A, Shipley P, et al. Randomized controlled trial of peer-led recovery education using Building Recovery of Individual Dreams and Goals through Education and Support (BRIDGES). Schizophr Res. 2012;136(1–3):36–42.

17. Pickett SA, Diehl SM, Steigman PJ, Prater JD, Fox A, Shipley P, et al. Consumer empowerment and self-advocacy outcomes in a randomized study of peer-led education. Community Ment Health J. 2012;48(4):420–30.

18. Eisen S V., Schultz MR, Mueller LN, Degenhart C, Clark JA, Resnick SG, et al. Outcome of a randomized study of amental health peer education and support group in the VA. Psychiatr Serv. 2012;63(12):1243–6.

19. Spaniol L, Koehler M, Hutchinson D. The recovery workbook: Practical coping and empowerment strategies for people with psychiatric disability. Boston: Center for Psychiatric Rehabilitation, Boston University, Sargent College of Allied Health Professions; 1994.

20. Kaplan K, Salzer MS, Solomon P, Brusilovskiy E, Cousounis P. Internet peer support for individuals with psychiatric disabilities: A randomized controlled trial. Soc Sci Med [Internet]. 2011;72(1):54–62. Available from: http://dx.doi.org/10.1016/j.socscimed.2010.09.037

21. Klaghofer R, Brähler E. Construction and statistical test of a shortform of the SCL-90-R. Psychiatr und Psychother. 2001;49:115–24.

22. Beck A, Steer R, Brown G. Manual for the Beck Depression Inventory–II. San Antonio: Psychological Corporation; 1996.

23. Corrigan PW, Salzer M, Ralph RO, Sangster Y, Keck L. Examining the factor structure of the recovery assessment scale. Schizophr Bull. 2004;30(4):1035–41.

24. Derogatis L, Spencer M. The Brief Symptom Inventory (BSI): Administration, scoring, and procedures manual – 1. Baltimore: Johns Hopkins University School of Medicine, Clinical Psychometrics Research Unit; 1982.

25. Snyder CR, Harris C, Anderson JR, Holleran SA, Irving LM, Sigmon ST, et al. The Will and the Ways: Development and Validation of an Individual-Differences Measure of Hope. J Pers Soc Psychol. 1991;60(4):570–85.

26. Brashers DE, Haas SM, Neidig JL. The patient self-advocacy scale: Measuring patient involvement in health care decision-making interactions. Health Commun. 1999;11(2):97–121.

27. Skevington S, Lotfy M, O’Connell K. The World Health Organization’s WHOQOL-BREF quality of life assessment: psychometric properties and results of the international field trial. Qual Life Res. 2004;13(2):299–310.

28. Giffort D, Schmook A, Woody C, Volendorf C, Gervain M. Construction of a scale to measure consumer Recovery. Springfield: Office of Mental Health.; 1995 p.

29. Pickett SA, Diehl S, Steigman PJ, Prater JD, Fox A, Cook JA. Early outcomes and lessons learned from a study of the Building Recovery of Individual Dreams and Goals through Education and Support (BRIDGES) program in Tennessee. Psychiatr Rehabil J. 2010;34(2):96–103.

30. Rogers S, Chamberlin J, Ellison M, Crean T. A consumer-constructed scale to measure empowerment among users of mental health services. Psychiatr Serv. 1997;48(8):1042–7.

31. Selim A, Rogers W, Fleishman J, Qian S, Fincke B, Rothendler J, et al. Updated US population standard for the Veterans RAND 12-item Health Survey (VR-12). Qual Life Res. 2009;18(1):434–52.

32. Eisen S V., Normand SL, Belanger AJ, Spiro A, Esch D. The revised behavior and symptom identification scale (BASIS-R): Reliability and validity. Med Care. 2004;42(12):1230–41.

33. Lehman A. A Quality of Life Interview for the chronically mentally ill. Eval Program Plann. 1988;11(1):51–62.

34. Derogatis L, Lipman R, Rickels K, Uhlenhuth E, Covi L. The Hopkins Symptom Checklist (HSCL): a self-report symptom inventory. Behav Sci. 1974;19(1):1–15.

35. Sherbourne C, Stewart A. The MOS social support survey. Soc Sci Med. 1991;32(6):705–14.

36. Eaton W, Smith C, Ybarra M, Muntaner C, Tien A. Center for Epidemiologic Studies Depression Scale: review and revision (CESD and CESD-R). In: Maruish M, editor. The Use of Psychological Testing For Treatment Planning and Outcomes Assessment: Volume 3: Instruments For Adults, 3rd edition. Mhwah (NJ); 2004. p. 363–77.

37. Corrigan PW, Michaels PJ, Vega E, Gause M, Watson AC, Rüsch N. Self-stigma of mental illness scale-short form: Reliability and validity. Psychiatry Res. 2012;199(1):65–9.

38. Ritsher J, Otilingam P, Grajales M. Internalized stigma of mental illness: psychometric properties of a new measure. Psychiatry Res. 2003;121(1):31–49.

39. Rüsch N, Corrigan P, Wassel A, Michaels P, Olschewski M, Wilkniss S, et al. A stress-coping model of mental illness stigma: I. Predictors of cognitive stress appraisal. Schizophr Res. 2009;110(1–3):59–64.

40. Russinova Z, Rogers ES, Gagne C, Bloch P, Drake KM, Mueser KT. A Randomized Controlled Trial of a Peer-Run Antistigma Photovoice Intervention. Psychiatr Serv. 2014;65(2):242–6.

41. Radloff L. The CES-D Scale: A Self-Report Depression Scale for Research in the General Population. Appl Psychol Meas. 1977;1(3):385–401.

42. Schwarzer R, Jerusalem M. Generalized Self-Efficacy scale. In: Weinman J, Wright S, Johnston M, editors. Measures in health psychology: A user’s portfolio Causal and control beliefs . Windsor: Nfer-Nelson; 1995. p. 35–7.
