# Supplementary material for: A systematic review and meta-analysis of group peer support interventions for people experiencing mental health conditions
Source: BMC Psychiatry. 2021 Jun 23;21:315. doi: 10.1186/s12888-021-03321-z (PMC8220835; doi:10.1186/s12888-021-03321-z)
Supplement: Supplementary file 3 — Additional file 3. [file 12888_2021_3321_MOESM3_ESM.docx]

Additional records identified through other sources
(n = 225)

Forward citation, n = 214; records within systematic reviews returned by database search, n = 9; sent by author, n = 1; Main trial search from protocol retrieved by the electronic search, n = 1.

Records identified through database searching
(n = 7,153 )

## Identification

Records after duplicates removed
(n = 4,277)

## Screening

Records screened
(n = 4,277)

Records excluded
(n = 4,179 )

Full-text articles excluded, with reasons
(n = 87)

Health professional involvement: 23

Complex intervention: 14

Wrong study design: 12

Protocol: 8

Individual peer support: 9

Not solely delivered to people with confirmed mental health conditions: 10

Duplicate: 1

Results not available at time of review: 2

Wrong outcomes: 4

Focus was not recovery: 1

Lived experience not a facilitator requirement: 1

Further clarification sought from authors but not received: 2

Full-text articles assessed for eligibility
(n = 98 )

## Eligibility

Studies included

(n = 8 )

8 trials reported by 11 publications

Studies included in quantitative synthesis
(n = 6)

6 trials reported by 8 publications

## Included
